# Molecular Beam Epitaxy Growth of Cadmium Telluride Structures on Hexagonal Boron Nitride

**DOI:** 10.1021/acsomega.3c05699

**Published:** 2023-11-14

**Authors:** Adam Krzysztof Szczerba, Julia Kucharek, Jan Pawłowski, Takashi Taniguchi, Kenji Watanabe, Wojciech Pacuski

**Affiliations:** † Faculty of Physics, University of Warsaw, Pasteura St. 5, Warsaw 02-093, Poland; ‡ Research Center for Materials Nanoarchitectonics, National Institute for Materials Science, 1-1 Namiki, Tsukuba 305-0044, Japan; § Research Center for Electronic and Optical Materials, National Institute for Materials Science, 1-1 Namiki, Tsukuba 305-0044, Japan

## Abstract

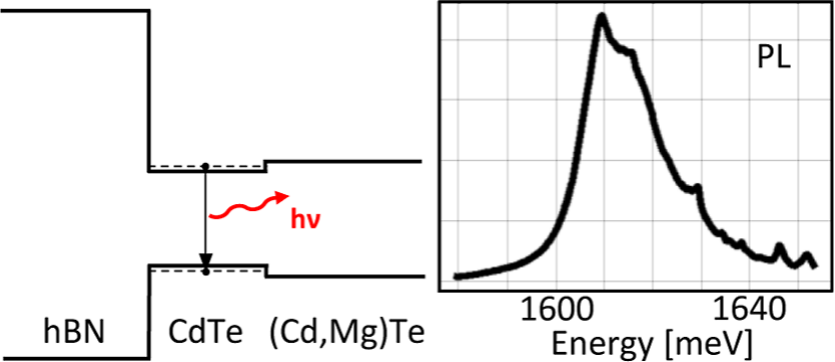

We investigate the feasibility of the epitaxial growth of a three-dimensional semiconductor on a two-dimensional substrate. In particular, we report for the first time the molecular beam epitaxy growth of cadmium telluride (CdTe) quantum wells on hexagonal boron nitride (hBN). The presence of the quantum wells is confirmed by photoluminescence measurements conducted at helium temperatures. Growth of the quantum wells on two-dimensional, almost perfectly flat hBN appears to be very different from growth on bulk substrates; in particular, it requires 70–100 °C lower temperatures.

## 1 Introduction

Hexagonal boron nitride (hBN) is a semiconductor with a very high band gap of approximately 5.8 eV^1,2^ and ultralow roughness when it is in the form of two-dimensional flakes exfoliated from high-quality bulk, such as bulk grown using high-pressure method.^3^ These properties make hBN an ideal substrate for epitaxial growth, which has been shown for the two-dimensional materials, such as graphene^4^ or transition metal dichalcogenides (TMDs) like WS_2_^5^ MoS_2_,^6^ MoSe_2_,^7,8^ and MoTe_2_.^9^ In particular, growth on hBN is instrumental for obtaining narrow excitonic lines of TMD monolayers^8^ without any mechanical postprocessing. Moreover, the high bandgap of hBN gives the possibility of using this material as a barrier in quantum structures.

The main goal of this work was to verify the effectiveness of growing three-dimensional semiconductors on hBN. Specifically, we decided to grow on hBN CdTe quantum wells (QWs) with (Cd,Mg)Te barrier on top because optical properties of CdTe/(Cd,Mg)Te QWs are extremely sensitive to the quality of the substrate^10^ and to growth conditions. Additionally, in a proposed configuration, the CdTe layer is in direct contact with hBN, revealing the quality of the hBN/CdTe interface. To our knowledge, this is the first report on the II–VI semiconductor structure grown on hBN.

## 2 Methods

Growth was performed using molecular beam epitaxy (MBE) in the growth chamber model SVT-35 placed at the University of Warsaw. To grow the samples, low-temperature effusion cells with Cd (7N purity), Mg (6N purity), and Te (7N purity) were used. The evolution of the surface during the growth process was observed with reflection high-energy electron diffraction (RHEED). With this method, we were able to distinguish between the situation when the hBN surface is covered by deposited material and the situation when efficient desorption leads to clean hBN despite exposure to molecular fluxes. Additionally, we observed that the exposition of the substrate on the electron beam slightly affects the growth conditions, as described in the Results and Discussion section. After growth, the sample surface was imaged using optical microscopy and atomic force microscopy (AFM). The presence of the QWs was verified through photoluminescence (PL) measurements conducted at a temperature of 10 K under a microscope objective with a laser spot of about 1 μm diameter. A laser with a wavelength of 445 nm was used to excite the samples. This wavelength corresponds to a photon energy of 2.8 eV, so it is sufficient to excite the valence band electrons to the conduction band of CdTe, considering that the band gap of CdTe at 10 K is 1.6 eV.^11^ The excitation power was relatively low (300 μW) to avoid the structural influence of the laser beam on the studied structure.

## 3 Design of the QW Structure on hBN

**Figure 1:**
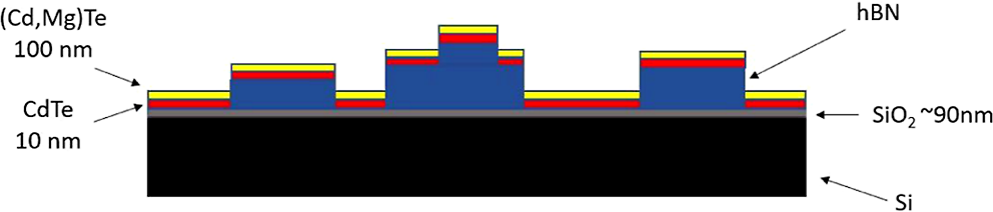
Scheme of the CdTe/(Cd,Mg)Te QW sample grown by MBE on hBN flakes exfoliated on a Si(100) wafer covered with 90 nm of SiO_2_. The thickness of CdTe is 10 nm, the thickness of (Cd,Mg)Te is 100 nm, and the thickness of hBN flakes varies between a few nm and a few hundreds of nm, typically about 100 nm.

Figure 1 illustrates the design of the samples investigated in this work. The substrates were prepared by exfoliating hBN flakes onto a semiinsulating, 10 mm large Si(100) wafer with 90 nm of SiO_2_. The bulk hBN used during the experiment was a high-quality material grown in the laboratory of Taniguchi and Watanabe.^3^ The exfoliated hBN flakes had an average thickness of approximately 100 nm and a typical size of tens of micrometers. On such substrate, a layered structure was grown through MBE, involving the growth of nominally 10 nm of CdTe as well as 100 nm of a barrier material, primarily (Cd,Mg)Te with about 10% of Mg. Since hBN flakes were covering SiO_2_ only partially, CdTe structures have been deposited at the same time on both hBN and SiO_2_.

**Figure 2:**
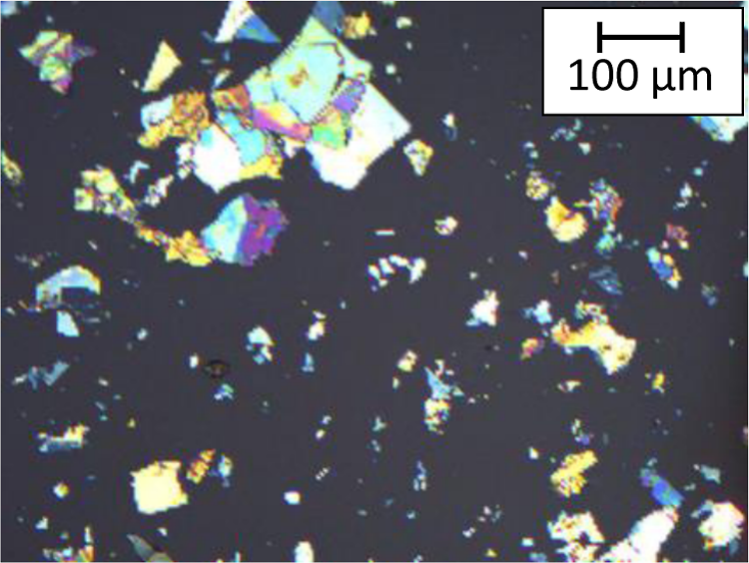
Example optical image of Si(100) substrate covered with 90 nm of SiO_2_ and exfoliated hBN flakes with various thicknesses and lateral size. Such substrates were further used for epitaxial growth of II–VI structures.

Optical images of part of the substrate’s surface are presented in Figure 2. Many hBN flakes with different shapes, sizes, and colors are visible. The color of the flake corresponds to its thickness, which means that each flake has a different height (from a few to hundred nanometers). Boron nitride flakes also have a huge range of lateral size, up to hundreds of micrometers.

**Figure 3:**
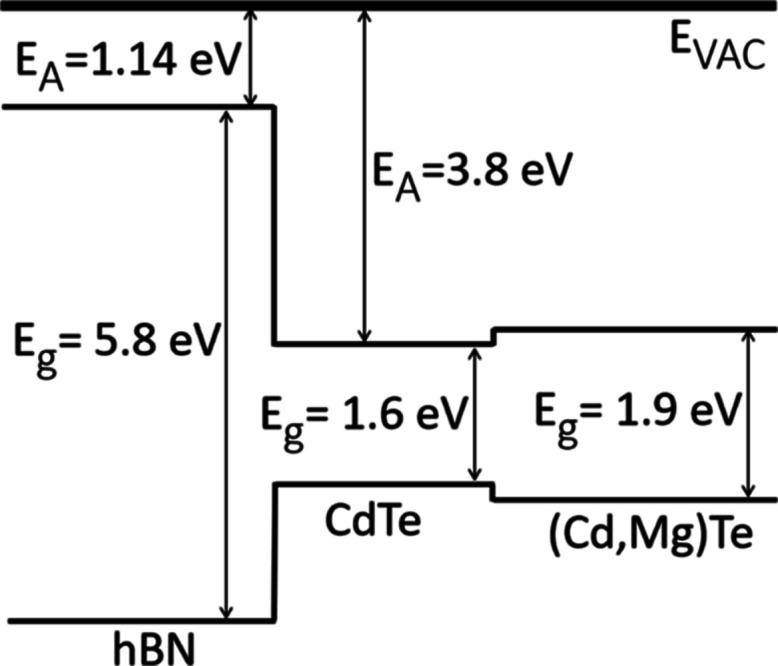
Scheme of the band structure of CdTe/(Cd,Mg)Te QW grown on hBN. *E*_VAC_ is a vacuum level, *E*_A_ is electron affinity, and *E*_g_ is energy gap of the used materials.

Band structure of CdTe/(Cd,Mg)Te grown on hBN is presented in Figure 3. Both hBN/CdTe and CdTe/(Cd,Mg)Te interfaces are I-type heterojunction, which means that the QW made of these materials is also I-type, and its optical properties are promising. Valence band offset α was calculated by dividing the valence band energy level *E*_v_ difference of both materials *X* and *Y* by band gap energy *E*_g_ difference^10^
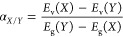


The valence band offset of hBN and CdTe was estimated based on the electron affinity for these materials.^12,13^ We estimate the valence band offset of CdTe and hBN to be approximately α_CdTe/hBN_ = 0.37. Valence band offset of CdTe and (Cd,Mg)Te is α_CdTe/(Cd,Mg)Te_ = 0.45.^14^

## 4 Results and Discussion

The technological experiment was started by calibration of growth rates (approximately 0.1 nm/s) using in situ optical reflectivity during growth of standard CdTe and (Cd,Mg)Te layers on a GaAs(100) substrate.^15^ Then, we started the growth of similar structures on hBN/SiO_2_/Si substrates, at the same temperature, which was equal to 320 °C. However, neither RHEED observation nor post growth optical and AFM imaging revealed the presence of the deposited material. We concluded that at such a temperature, the sticking coefficient is zero for perfectly flat surfaces. There was some material grown on SiO_2_ on part of the sample, but even on hBN flakes surrounded by grown material on SiO_2_, there was no material observed. Consequently, we substantially decreased the substrate temperature during the growth of the next samples.

**Figure 4:**
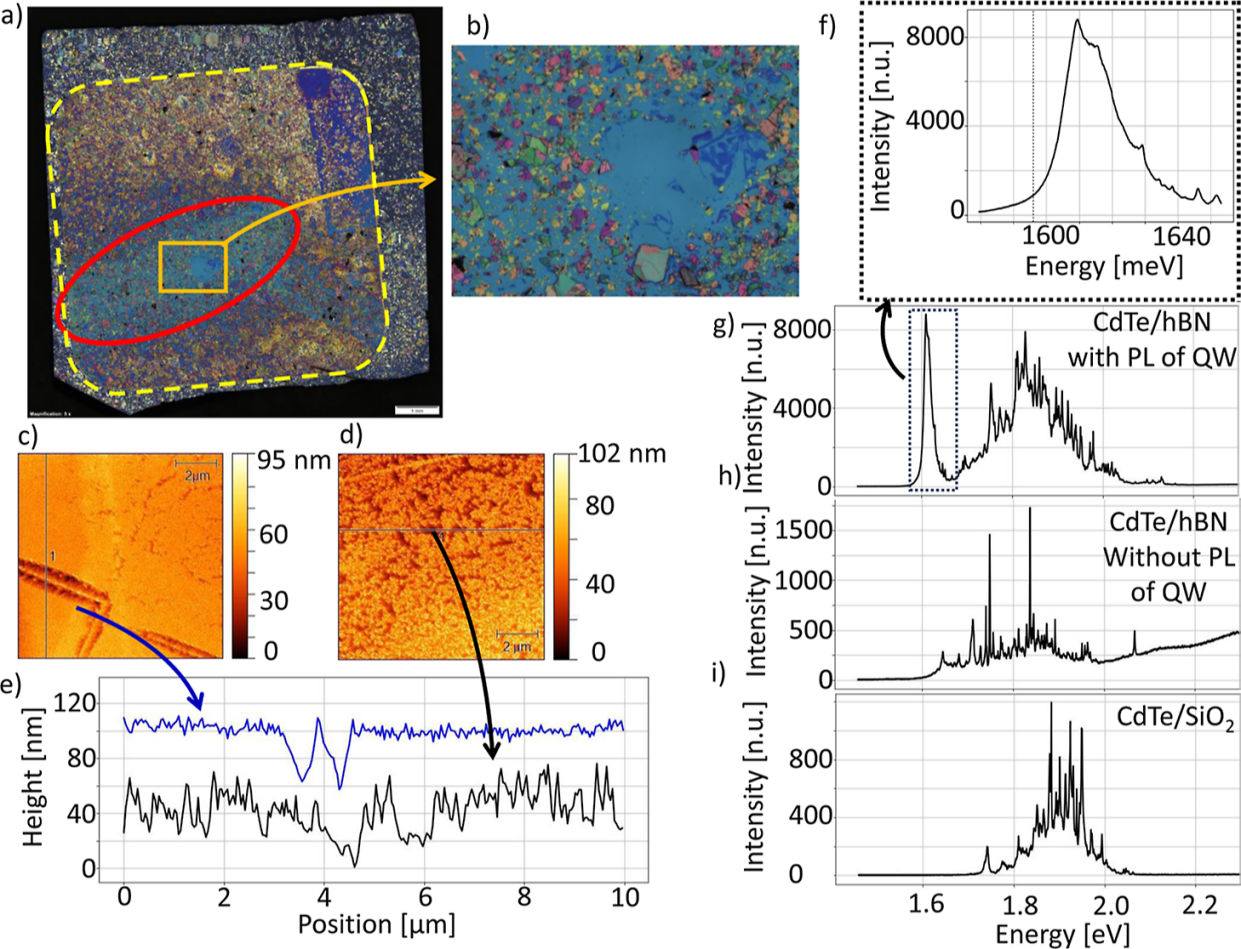
Sample with CdTe QW deposited at 250 °C on hBN flakes exfoliated on Si, all covered with (Cd,Mg)Te barrier. (a) Optical image of the whole 8.5 mm × 9 mm sample. The sample is covered by exfoliated hBN flakes, as in Figure 2. The growth has occurred only in the area marked by a dashed square in the middle of the sample, and the area outside the square was not exposed to the molecular fluxes. The area influenced by high-energy electrons from RHEED gun was marked by ellipsoid. (b) Magnification of part of the sample marked in (a). In the middle of the image, the area without exfoliated hBN is visible. This area is surrounded by many hBN flakes of different sizes and colors. (c) AFM scan of material grown on hBN in an area influenced by RHEED. (d) AFM scan of material grown on hBN in the area without the influence of RHEED. (e) Comparison of roughness of the material grown on hBN in the area influenced by RHEED (blue line, rms = 3.426 nm) and in the area without such influence (black line, rms = 6.1 nm), (f) magnification of PL spectrum presented in (g) with CdTe QW signal. (g) Broad-range PL spectrum of the structure measured at 10 K. A strong peak in characteristic energy close to 1610 meV is identified as related to CdTe QW. Multiple peaks appearing in a wide range of energies were associated with the PL signal of the barrier. (h) PL spectrum of the structure in areas where the presence of CdTe QW is not evident. Multiple peaks that appeared in a wide range of energies were associated with the PL signal of the barrier. (i) Typical PL spectrum of the structure grown in the same process but on SiO_2_. Multiple peaks that appeared in a wide range of energies were associated with the PL signal of the barrier.

The sample presented in Figure 4 was grown at a temperature of 250 °C, which is approximately 70 °C lower than the growth temperature typically used for the growth of CdTe QWs on bulk substrates. Optical images (Figure 4a,b) of the sample reveal a high number of hBN flakes in the whole area of the sample. Almost whole of the sample is covered by the deposited layer, except edges which were not exposed to molecular fluxes. The observed blue-violet color of the deposited layer results from optical interferences. Subtle changes in the color is a consequence of small differences in layer thickness and the resulting optical interferences. They reveal in particular an area marked by red ellipsoid, which was exposed to an electron beam related to RHEED measurements during growth without substrate rotation. AFM was used to scan the surface of the material grown on hBN on different regions on the sample. In particular, AFM reveals a difference between areas which are affected by electron beam (Figure 4c) and other areas (Figure 4d).

(Cd,Mg)Te grown on hBN appears to be more compact in the region influenced by electrons. Cross-sections of AFM scans in Figure 4c,d are presented in Figure 4e. In the area influenced by RHEED, the typical height difference is approximately 10 nm; however, 40 nm deep valleys, caused by atomic steps on the substrate, are visible. Cross-section of the area without electron beam influence presents huge height differences in short distance on the sample. The largest difference in this graph is approximately 80 nm in range of 1 μm, which is 80% of nominal thickness of (Cd,Mg)Te barrier. Representative PL spectra measured in various spots of the sample are shown in Figure 4f–h for the structure grown on hBN and in Figure 4i for the structure grown on SiO_2_.

The typical PL signal of the CdTe QW grown on hBN is shown in Figure 4g, with an intense peak located at 1610 meV, which is a typical spectral position for CdTe QWs grown on bulk substrates, only slightly blue-shifted from position known for bulk, 1596 meV,^10^ what is visible in Figure 4f. Interestingly, such PL spectra are observed mainly in the area of the sample, which was affected by RHEED observations. Figure 4h shows areas where PL of CdTe QW on hBN is less evident; it is at higher energy (position 1645 meV)that the intensity is weaker, and the peak is merged with an ensemble of sharp lines. Emission energy of the QW strongly depends on the thickness of the QW, but can be also increased by interdiffusion of Mg from (Cd,Mg)Te barrier. In our case, both reasons can be responsible for the observation of the first peak at higher energy than usual, as is shown in Figure 4h.

**Figure 5:**
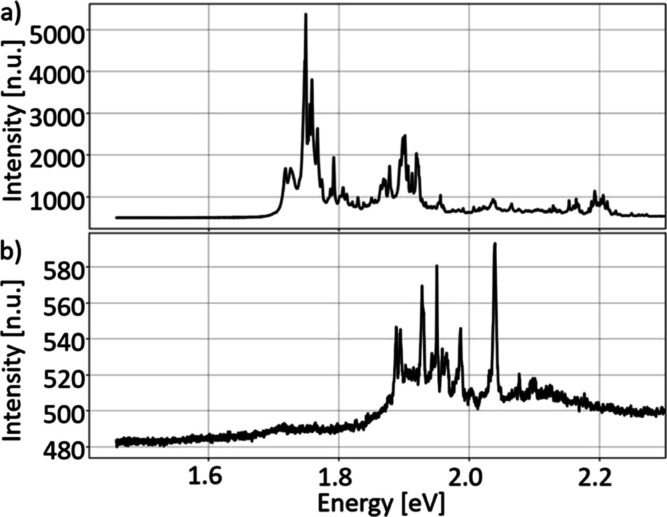
PL spectra of a reference (Cd,Mg)Te layer, without a QW, measured at a temperature of 10 K. Multiple peaks appearing in a wide range of energies were associated with the PL signal of the barrier. (a) Typical PL spectrum of 100 nm (Cd,Mg)Te grown on hBN. (b) Typical PL spectrum of 100 nm (Cd,Mg)Te grown on SiO_2_.

In Figure 4i, for the structure grown on SiO_2_, there is no clear peak close to the expected emission energy of CdTe QWs; only an ensemble of sharp lines is observed. Similar PL spectrum containing many sharp lines in the range 1.7–2.05 eV was observed for all areas of the sample. In order to explain the origin of multiple lines observed in a wide range, we have grown and studied a reference sample where only the (Cd,Mg)Te barrier was deposited, without CdTe QW. The PL of such a reference sample is shown in Figure 5a for (Cd,Mg)Te deposited on hBN, and in Figure 5b for (Cd,Mg)Te deposited on SiO_2_. In both cases, multiple sharp lines in a wide range are observed. This indicates that such sharp lines are not related to CdTe QW, and they are related just to the (Cd,Mg)Te barrier. Lines of (Cd,Mg)Te observed at various energies indicate a high structural and compositional disorder of this material. This is consistent with the results obtained for bulk (Cd,Mg)Te and epitaxial (Cd,Mg)Te on 3D substrates, where the tendency for separation of various phases is observed.^16,17^ Based on the emission energy in the wide range between 1.7 and 2.2 eV (e.g., Figure 5a), composition in various grains corresponds to Mg concentration between 5 and 30%.

**Figure 6:**
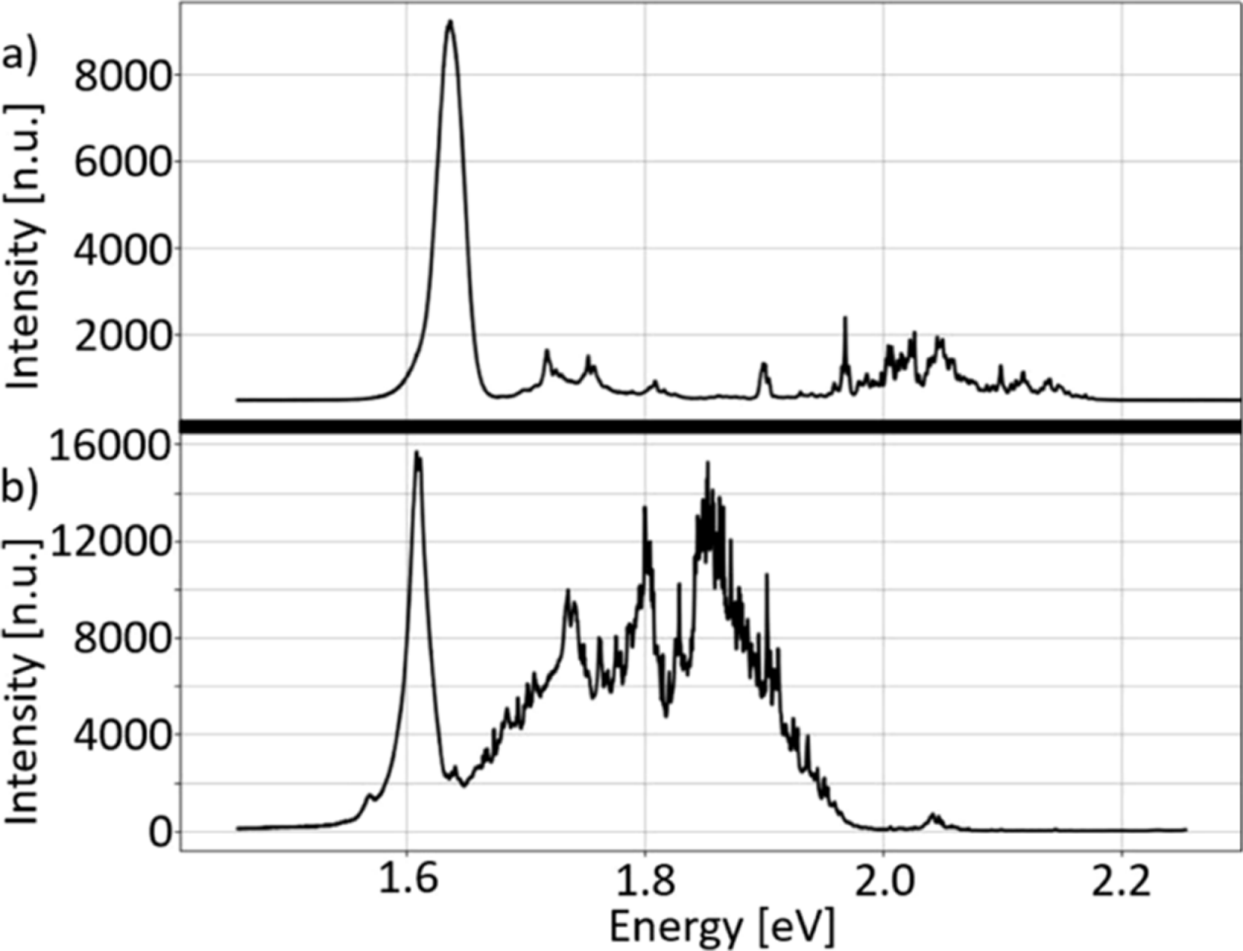
(a) Typical PL spectra of 10 nm CdTe and 100 nm (Cd,Mg)Te grown on hBN in temperature 100 °C lower than the growth temperature on a three-dimensional material. Multiple peaks that appeared in a wide range of energies were associated with the PL signal of the barrier. (b) Typical PL spectra of 10 nm CdTe and 100 nm (Cd,Mg)Te grown on hBN annealed before the growth. Multiple peaks that appeared in a wide range of energies were associated with the PL signal of the barrier.

Throughout this experiment, multiple samples were grown to identify the best conditions for the growth. As a result, it was found that the optimal substrate temperature during growth was 220 °C, which is approximately 100 °C lower than the growth temperature on the three-dimensional material. The typical PL spectrum of CdTe QW grown on hBN in these conditions is illustrated in Figure 6a. The observed peak of QW is much stronger than in the case of the first grown sample presented in Figure 2a, grown at 250 °C.

In order to understand the role of electron beam in the formation of QWs, we performed experiments with high-temperature (800 °C) annealing of the substrate before the growth of QWs. In such structures, there were no traces of electron beam anymore, and QWs were observed in the whole area where CdTe/(Cd,Mg)Te was deposited on hBN (Figure 6b). Therefore, the electron beam acts in a similar way as degassing at high temperature. Another conclusion is that degassing the substrate at about 200 °C just before the growth is not enough to clean the surface properly.

**Figure 7:**
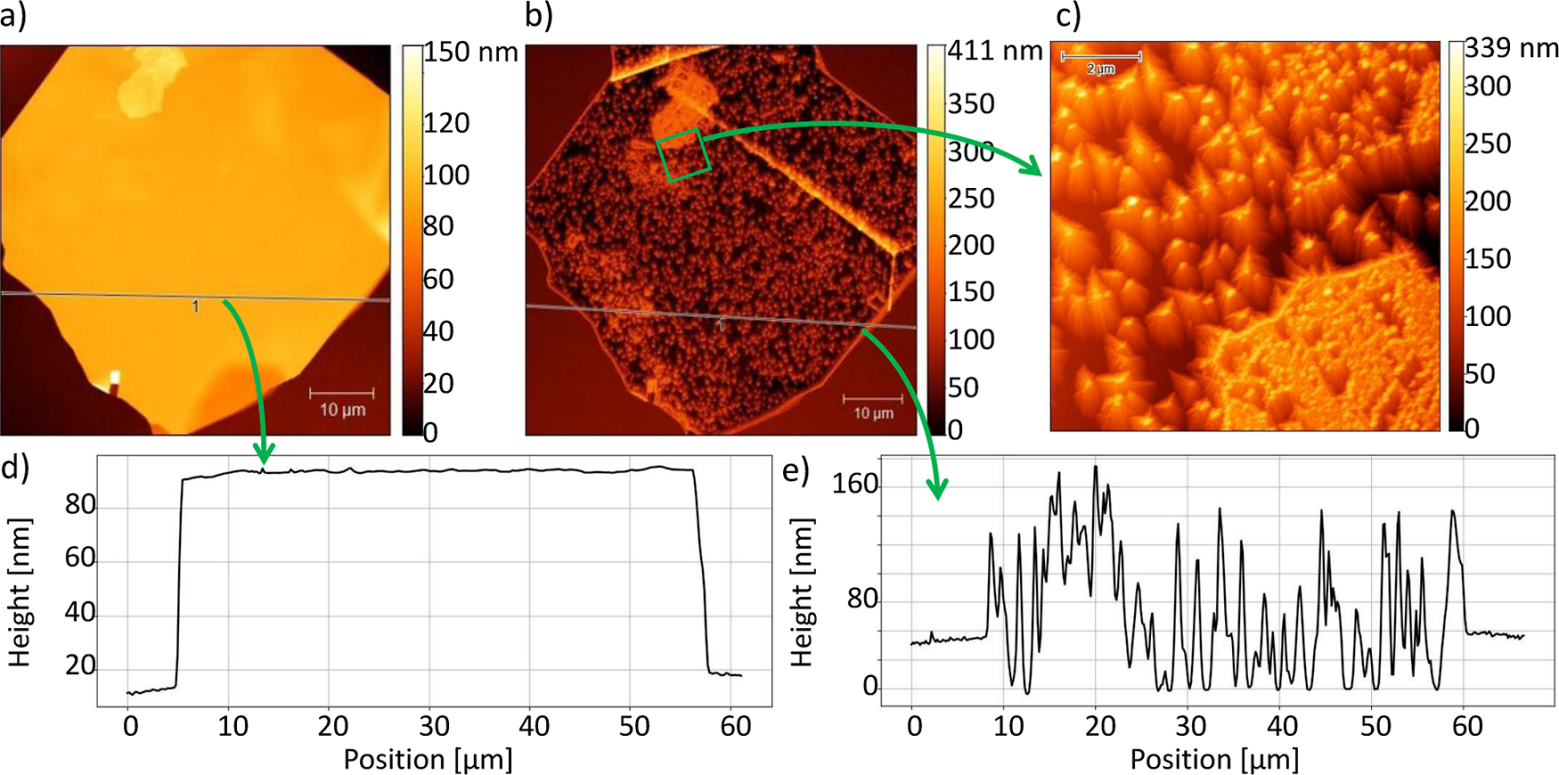
AFM scans and cross-sections of the hBN flake before and after the growth of 10 nm CdTe and 100 nm (Cd,Mg)Te performed at 220 °C on the substrate annealed at 800 °C before the growth process. (a) AFM scan of hBN flake before the growth. (b) AFM scan of hBN flake after the growth. The evolution of the flake’s substrate, in comparison to the image shown in (a) is visible, which means that growth has occurred. (c) AFM scan of part of the surface of the material grown on hBN [marked by a square in (b)]. Many structures similar to triangle-based pyramids are visible, indicating the (111) crystal orientation of the growth. (d) Cross-section of the AFM scan of the hBN flake before the growth was performed along the line marked in (a). The flake has a height of 80 nm, and the root mean square(rms) calculated on hBN along the line was approximately 58.49 pm. (e) Cross-section of the AFM scan of the material grown on hBN performed along the line marked in (b). The height levels of the material grown on SiO_2_ and on hBN are similar, which indicates different conditions of growth on both surfaces. rms of the material grown on hBN calculated along the line is approximately 12.06 nm.

AFM scans of the sample of 10 nm CdTe and 100 nm (Cd,Mg)Te grown in the best conditions (growth performed in 220 °C on the substrate annealed in 800 °C) and scan of the same flake before the growth are shown in Figure 7a–c. The evolution of the surface of the sample is clearly visible. The cross-section of the hBN flake before and after the growth is presented in Figure 7d,e.

On the cross-section presented in Figure 7d, the thickness of the hBN flake before growth was determined to be approximately 80 nm. The rms of the hBN flakes along the cross-section line is 58.49 pm. After the growth on the hBN, structures similar to pyramids are visible, and the rms on the sample along the cross-section line is approximately 12.06 nm. Material grown on SiO_2_ is denser (rms = 7.94) than the material on hBN. Furthermore, the height level of the material grown on SiO_2_ is similar to the height level of the material on hBN, which shows how the growth conditions in both areas are different.

Many pyramid structures visible in Figure 7c have a triangular base. This observation indicates that trigonal symmetry of the substrate resulted in the growth of CdTe and (Cd,Mg)Te with the same symmetry, therefore in the (111) crystallographic direction.

**Figure 8:**
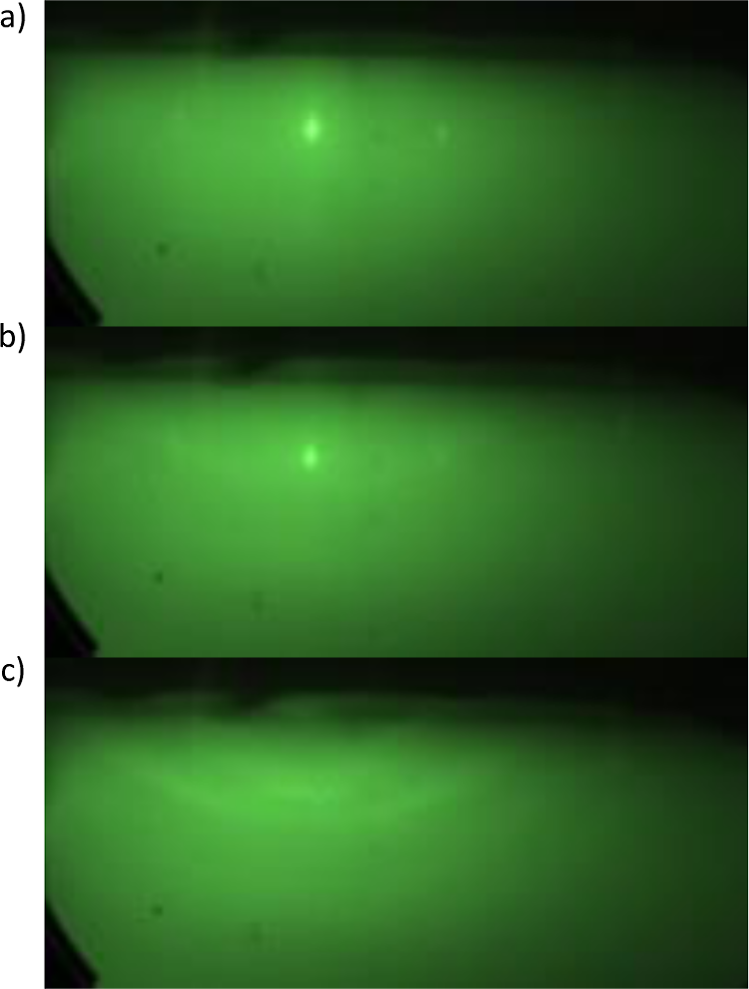
RHEED images obtained during the growth at 220 °C on the substrate annealed at 800 °C in various stages. (a) hBN substrate before growth. (b) hBN with nominally 10 nm of CdTe. (c) Completed CdTe/(Cd,Mg)Te structure. Rings indicate the polycrystalline structure.

During the growth of the samples, the changes on the surface were observed with a RHEED signal. Example images of such measurements are presented in Figure 8, for the growth performed at 220 °C on the substrate annealed at 800 °C before the growth process. The well-defined diffraction pattern on the image Figure 8a originates from the hBN surface, while scattering from SiO_2_ contributes to the background. After the growth of the nominally 10 nm thick CdTe layer, the signal of hBN is weakening, and the deposited material appears as a delicate ring. After the growth of the whole sample, the hBN signal completely disappears, and many rings are visible. This indicates that the whole hBN was covered by polycrystalline material.

About a 10 nm thick layer of CdTe should form a structure, in which the quantum effects are significant vertically and neglectable laterally. Therefore, such a structure can be considered as a QW. Moreover, the observed emission energy, about 1610 meV, agrees well with the characteristic emission energy of CdTe QWs grown on other substrates.^14^

## 5 Conclusions

Several CdTe QWs on hBN samples were grown using MBE with lowering of substrate temperature compared to that for growth on bulk substrates. The first QW was detected on the sample grown at a temperature approximately 70 °C lower than the typical growth temperature typically used for bulk materials. The typical PL signal of CdTe QW deposited directly on hBN is observed close to 1610 meV, similarly to well-known CdTe/(Cd,Mg)Te QWs. Furthermore, the surface’s structure of (Cd,Mg)Te grown on hBN was analyzed through AFM scans. The properties of the barrier material were found to be connected with the broad PL spectra presented in Figure 5a,b. This spectra appears in all PL signals of the samples with the (Cd,Mg)Te barrier, as shown in Figures 4f–h and 6a–c.

The optimal temperature of the substrate was found to be 220 °C, which is approximately 100 °C lower than the growth temperature on the three-dimensional material. In this growth temperature, the PL signal of CdTe QW (Figure 6a) was observed over the majority of the hBN. For effective growth of CdTe QWs on hBN, the substrate should be influenced by high-energy electron beam or preheated to 800 °C before the growth.

The studied hBN/CdTe/(Cd,Mg)Te heterostructure appears to be a new, high optical quality type-I QW that benefits from the ultrahigh flatness of 2D barrier. This opens an exciting possibility of redesigning various QWs systems by replacing the bottom barrier with hBN.
